# Correction: Kopecka et al. Insights into P-Glycoprotein Inhibitors: New Inducers of Immunogenic Cell Death. *Cells* 2020, *9*, 1033

**DOI:** 10.3390/cells15100866

**Published:** 2026-05-09

**Authors:** Joanna Kopecka, Martina Godel, Silvia Dei, Roberta Giampietro, Dimas Carolina Belisario, Muhlis Akman, Marialessandra Contino, Elisabetta Teodori, Chiara Riganti

**Affiliations:** 1 Department of Oncology, University of Torino, via Santena 5/bis, 10126 Torino, Italy; joanna.kopecka@unito.it (J.K.); martina.godel@edu.unito.it (M.G.); dimascarolina.belisario@unito.it (D.C.B.); muhlis.akman@unito.it (M.A.); 2 Department of Neurosciences, Psychology, Drug Research and Child Health, Section of Pharmaceutical and Nutriceutical Sciences, University of Firenze, via Ugo Schiff 6, 50019 Sesto Fiorentino, Italy; silvia.dei@unifi.it (S.D.); elisabetta.teodori@unifi.it (E.T.); 3 Department of Pharmacy-Pharmaceutical Sciences, University of Bari, via Orabona 4, 70125 Bari, Italy; giampietroroberta@gmail.com (R.G.); marialessandra.contino@uniba.it (M.C.)

In the original publication [1], there was a mistake in Figure 5B as published. During figure assembly, the panel corresponding to the condition “MDA/DX R-3” of Figure 3A was erroneously mounted as the panel corresponding to the condition “- dox KO#2” of Figure 5B. The corrected Figure 5 appears below. 

The authors state that the scientific conclusions are unaffected. This correction was approved by the Academic Editor. The original publication has also been updated.

## Figures and Tables

**Figure 5:**
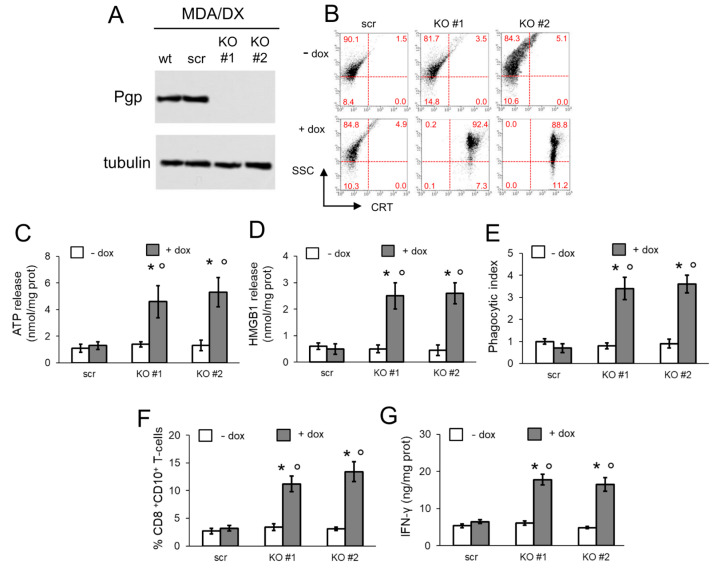
Pgp knock-out restores doxorubicin-induced immunogenic cell death. MDA-MB-231/DX cells (wild-type, wt) were transduced with a non-targeting vector (scrambled vector; scr) or with two CRISPR pCas Pgp-targeting vectors (KO#1, KO#2). Cells were grown in fresh medium (- dox) or in the presence of 5 μM doxorubicin (+ dox), for 6 h (**B**,**C**) or 24 h (**D**–**F**). (**A**). Pgp immunoblotting in whole cell extracts. Tubulin was used as control of equal protein loading. The image is representative of 1 out of 3 experiments. (**B**). Dot plots representative of surface calreticulin (CRT), performed by flow cytometry in duplicates (*n* = 3). Numbers represent the percentage of cells/quadrants. Cut-off for positivity was fixed at 102. SSC: side-scatter. The image is representative of 1 out of 3 experiments. (**C**). Release of extracellular ATP, measured by a chemiluminescence-based assay in duplicates (*n* = 3). Data are means ± SD. * *p* < 0.001: KO-cells vs. scr-cells; ° *p* < 0.001: + dox cells vs. - dox cells. (**D**) Release of extracellular HMGB1, measured by ELISA in duplicates (*n* = 3). Data are means ± SD. * *p* < 0.001: KO-cells vs. scr-cells; ° *p* < 0.001: + dox cells vs.-- dox cells. (**E**) After the treatment indicated in (**D**), tumor cells were stained with FITC-PKH2 dye, DCs were stained with an anti-PE-HLA-DR antibody. Tumor cells were co-incubated with DCs for 24 h. Double-stained cells were counted by flow cytometry. Data are means ± SD (*n* = 3). * *p* < 0.001: KO-cells vs. scr-cells; ° *p* < 0.001: + dox cells vs. - dox cells. (**F**) T-lymphocytes were co-cultured with DCs after phagocytosis, then incubated 24 h with scr- or KO-cells. The percentage of CD8^+^ CD107a^+^ T-cells was measured by flow cytometry. Data are means ± SD (*n* = 3). * *p* < 0.001: KO-cells vs. scr-cells; ° *p* < 0.001: + dox cells vs. - dox cells. (**G**) IFN-γ concentration was measured in the supernatants of T-lymphocytes, treated as indicated in (**F**), by ELISA in duplicates. Data are means ± SD (*n* = 3). * *p* < 0.001: KO-cells vs. scr-cells; ° *p* < 0.001: + dox cells vs. - dox cells.
